# Do Humidity and Temperature Impact the Spread of the Novel Coronavirus?

**DOI:** 10.3389/fpubh.2020.00240

**Published:** 2020-05-27

**Authors:** Shu Yuan, Si-Cong Jiang, Zi-Lin Li

**Affiliations:** ^1^College of Resources, Sichuan Agricultural University, Chengdu, China; ^2^Chengdu KangHong Pharmaceutical Group Comp. Ltd., Chengdu, China; ^3^Department of Cardiovascular Surgery, Xijing Hospital, Medical University of the Air Force, Xi'an, China

**Keywords:** SARS-CoV-2, temperature, humidity, virus transmission, environmental persistence

Since the outbreak of the novel SARS-like coronavirus (SARS-CoV-2) in Wuhan, China, more than 4,000,000 cases have been reported globally. Recently, a large number of reports have been published to discuss the possible roles of environmental factors in transmission of the novel coronavirus. They have focused especially on the impact of two air parameters, temperature and humidity, on the spread of SARS-CoV-2.

## Contradictory Reports About the Roles of Temperature and Humidity on SARS-CoV-2 Transmission

Various reports have drawn contradictory conclusions even when using the similar meteorological data and epidemic data collected in cities in China from January to April 2020. For example, Liu et al. (1) analyzed meteorological data of 30 cities in China and suggested that low temperature, mild diurnal temperatures, and low humidity likely aid the transmission of novel coronavirus disease 2019 (COVID-19). Shi et al. (2) also suggested that the incidence of COVID-19 decreases with an increase in temperature. Qi et al. (3) further showed that both temperature and humidity were negative associated with COVID-19. Using the similar meteorological data of 122 cities in China, however, Xie and Zhu (4) found no evidence to support that theory that the number of COVID-19 cases would reduce when the weather became warmer. They even showed a positive correlation between temperature and COVID-19 cases in that 1°C rise in the mean temperature (when <3°C) was associated with a 4.9% increase in the daily confirmed cases. Also, with meteorological data in China, Yao et al. (5) demonstrated that neither ambient temperature nor ultraviolet radiation has a significant impact on the transmission ability of SARS-CoV-2. For countries outside of China, there are also a large number of contradictory reports. Bashir et al. (6) found that average temperature, minimum temperature, and air quality were significantly associated with the COVID-19 pandemic in New York, USA, although the correlations were complex. In Jakarta, Indonesia, Tosepu et al. (7) demonstrated that the average temperature was indicated to be significantly correlated with the COVID-19 pandemic. In the city of Barcelona, Spain, Tobíasa and Molina (8) further indicated that a 1°C increase of max temperature reduced a decrease in the incidence rate by 7.5% on the same day. However, also in Spain, Briz-Redóna and Serrano-Arocab (9) found no significant relationship between COVID-19 cases and the temperatures. In addition, Jahangiri et al. (10) indicated that the transmission rate of the COVID-19 exhibited a low sensibility to the changes in the ambient temperature in Iran.

## Possible Reasons for the Contradictory Conclusions

What is the reason behind these contradictory findings? One reason is the different types of data included in the analyses. Some researchers collected counts of confirmed cases or new cases or total cases, but other researchers calculated the cumulative incidence rate. Different places (cities) have different population sizes and population densities. Considering that the population size significantly regulates the spread rate of COVID-19 (10), and the incidence rate per unit population may reflect the epidemics more accurately. The basic numbers of infected people in the center and border of epidemic areas are much different from those in areas of sporadic outbreak; that is to say, the infection probabilities of different areas are different. Therefore, it will be helpful to distinguish concentrated-outbreak areas with sporadic-outbreak areas when studying the role of environmental factors on COVID-19. For example, Shi et al. (2) counted confirmed cases in Wuhan and regions other than Wuhan in Hubei Province separately. (c) The time period selected for the research is also important. For example, after February 15, new cases in China have shown a downward trend and a gradual rise in temperature. We cannot thus conclude that the temperature was negatively correlated with new case numbers (1). There usually exits a bell curve in an epidemic. Researchers should focus on epidemic data and climate data only in the platform period ideally rather than in the exponential period or in the recession period. The platform period may provide the highest resolution data set for determining correlations between environmental conditions and COVID-19 transmission, and refraining from collection of data before and after would be irresponsible and could lead to missed opportunities to understand how outbreaks begin and resolve. The spread of the virus is also influenced by human factors, such as efficiency of case finding and contact tracing, quarantine strategy, implementation ability of COVID-19 control policy, urbanization rate, and availability of medical resources. How to normalize these complex epidemic data poses a great challenge to the researchers. The temperature and humidity ranges included in the current studies have often been small, such as the temperature range from 0 to 20°C in most China cities from January to March (1–5). The survivability of airborne viruses begins to decrease only when the temperature is more than 30°C (11, 12). Efficiency of the influenza virus transmission has been found to be dependent on relative humidity (RH) at 20°C but independent of air humidity at 30°C (11). The novel coronavirus may also apply to this rule. Maybe a global scale research will be able to obtain more accurate data of larger ranges of temperature and humidity.

## Weak Correlations Between Temperature and Humidity and COVID-19 Pandemic

Another reason for the opposite conclusions drawn from different researchers may be the weak correlations between temperature and humidity and SARS-CoV-2 propagation. For example, correlation coefficients acquired from the COVID-19 pandemic in New York, USA, were as low as 0.25–0.4 (6). The correlation coefficient between average temperature and COVID-19 pandemic in Jakarta, Indonesia, was only 0.392 (7). The high level of virus shedding and long viral persistence in aerosols may be main reasons. High levels of the virus in throat swabs have been observed in COVID-19 patients [either symptomatic or asymptomatic; (13, 14)]. It is worth noticing that the peak viral load of SARS-CoV-2 was more than 1,000 times higher than SARS-CoV-1, and active SARS-CoV-2 replication in upper respiratory tract tissues has been found, where SARS-CoV-1 is not thought to replicate at this site (13). The half-lives of SARS (SARS-CoV-1) and SARS-CoV-2 were almost the same in aerosols with averages of about 1.1–1.2 h (15), which is, however, about two times the half-life of influenza viruses [31.6 min; (16)]. Thus, for SARS-CoV-2, 1-h half-life might be enough long for an effective transmission, where temperature or humidity only exerts a week influence to the viral persistence.

## In Temperate Zone and Tropical Countries, Humidity May Play a More Important Role in Viral Transmission Than Temperature

For the influenza virus, at lower RH (<35%), the spreading efficiency was as high as that at a high RH (60–80%), while the spreading efficiency was at the lowest at moderate-range RH [e.g., 40–60%; (11)]. The influenza virus persistence presents an asymmetrical *U*-shaped curve at various RH at about 20°C (17). A 30-years county-level observation from the United States indicated that more than 50% of difference in the seasonal influenza mortalities may be attributed to air humidity alone; nevertheless, temperature may only impart a moderate influence (18).

Given that the pressure of saturation vapors is correlated with the temperature exponentially, both water vapor levels and temperatures influence RH. Therefore, either temperature or RH has effects on evaporation, thus influencing size of the droplets (12). The survival time of influenza virus in aerosol is prolonged at low RH, which may result in a low viral dose required for an efficient trans-infection. A large number of studies have suggested that both size distribution of droplets and the dynamic of the virus emitted through coughing are affected by air humidity (12, 19). As a general rule, in the temperate zone, low RH is closely related to the onset of the epidemic. While in tropical countries, humid-rainy conditions favor outbreaks (12, 19). High transmission ability of SARS-CoV-2 has been reported in south China cities (1, 4, 5) and Indonesia (7), both of which are humid-rainy warm regions.

It is suggested that, at a high humidity, such as humidity >70%, viruses survive under moist conditions in the droplets under physiological contents of salts; at moderate humidity (40–60%), salts are concentrated due to the evaporation that deactivates the viruses; and, at low humidity, humidity <30%, salts crystallize out of solution, which may keep the virus active (20). It is also proposed that, under dry but cool indoor environments, such as in winter, low humidity allows viral aerosols persist for a longer time in air because of their smaller sizes, thus increasing the spread of the virus. In warm areas, however, the relatively-low temperature and near-saturation humidity during the rainy season facilitate the spread of the virus via large aerosol particles (12). A recent study reported that a COVID-19 patient had transmitted SARS-CoV-2 to eight healthy contacts through bathing at a bath center, implying that the cluster spread of the virus could still arise in a condition of warm (hot) and near-saturation humidity (21). The viral transmission pattern under near-saturation humidity is rarely studied, highlighting a necessary area for future investigations.

## In Arid Areas, Cool, and Dry Conditions May Facilitate the Spread of the Virus

Contrasting with the high humidity areas, RHs of Iran, Iraq, India, the midwestern United States, and other arid areas in the middle and low latitudes are usually lower than 20%. Although the midday temperatures in these areas are usually >20°C (or even > 30°C), the night temperature may below 10°C (especially at high altitudes), which may facilitate the spread of the virus. The influenza virus can survive for more than 24 h in the environment of 23–25% RH and 7–8°C (22). Considering the longer half-life of SARS-CoV-2, the novel SARS-like coronavirus may survive for several days under a cool and arid condition. A recent report indicated that inhalation of dry air (20% RH, compared with 50% RH) impairs mucociliary clearance, the innate antiviral defense against influenza virus infections (23). In other words, the arid condition may not only increase the persistence of the viral but may also compromise the host's immunity.

## Prospective Comments

Although the viral transmission may be mainly determined by the humidity, the temperature may still impart a moderate influence on COVID-19 pandemic (1–5). Now, the virus has spread to the southern hemisphere, and these places are already experiencing autumn and are preparing for winter. The gradually decreased temperature may bring more difficulties to the epidemic control. Some areas of the southern hemisphere are very dry, such as the center of Australia and the interiors of Chile and Argentina. Humidifiers should be used at these places.

Most of the current studies have been based on the correlation analysis between the meteorological data and the epidemic data. However, direct studies with SARS-CoV-2 under different environmental conditions are still lacking. The environment variables determining the viral persistence and spread of SARS-like coronaviruses through aerial habitats are still less understood. Besides the studies of epidemiology, biological sciences and medical sciences, the contributions of environmental research are urgently warranted for controlling the spread of SARS-CoV-2 globally.

## Author Contributions

SY conceptualized the analysis and wrote the original draft. S-CJ and Z-LL reviewed and edited the manuscript. All authors have read and agreed to the published version of the manuscript.

## Conflict of Interest

S-CJ was employed by the Chengdu KangHong Pharmaceutical Group Comp. Ltd. The remaining authors declare that the research was conducted in the absence of any commercial or financial relationships that could be construed as a potential conflict of interest.
