# Corrigendum: Do Humidity and Temperature Impact the Spread of the Novel Coronavirus?

**DOI:** 10.3389/fpubh.2020.00317

**Published:** 2020-07-03

**Authors:** Shu Yuan, Si-Cong Jiang, Zi-Lin Li

**Affiliations:** ^1^College of Resources, Sichuan Agricultural University, Chengdu, China; ^2^Chengdu KangHong Pharmaceutical Group Comp. Ltd., Chengdu, China; ^3^Department of Cardiovascular Surgery, Xijing Hospital, Medical University of the Air Force, Xi'an, China

**Keywords:** SARS-CoV-2, temperature, humidity, virus transmission, environmental persistence

An author name was incorrectly spelled as **S-Cong Jiang**. The correct spelling is **Si-Cong Jiang**. The authors apologize for this error and state that this does not change the scientific conclusions of the article in any way. The original article has been updated.

